# Supplementary material for: Intracellular Survival of Biofilm-Forming MRSA OJ-1 by Escaping from the Lysosome and Autophagosome in J774A Cells Cultured in Overdosed Vancomycin
Source: Microorganisms. 2022 Feb 2;10(2):348. doi: 10.3390/microorganisms10020348 (PMC8874447; doi:10.3390/microorganisms10020348)

Figure S1. Morphological alterations of J774A with/without OJ-1 ingestion.

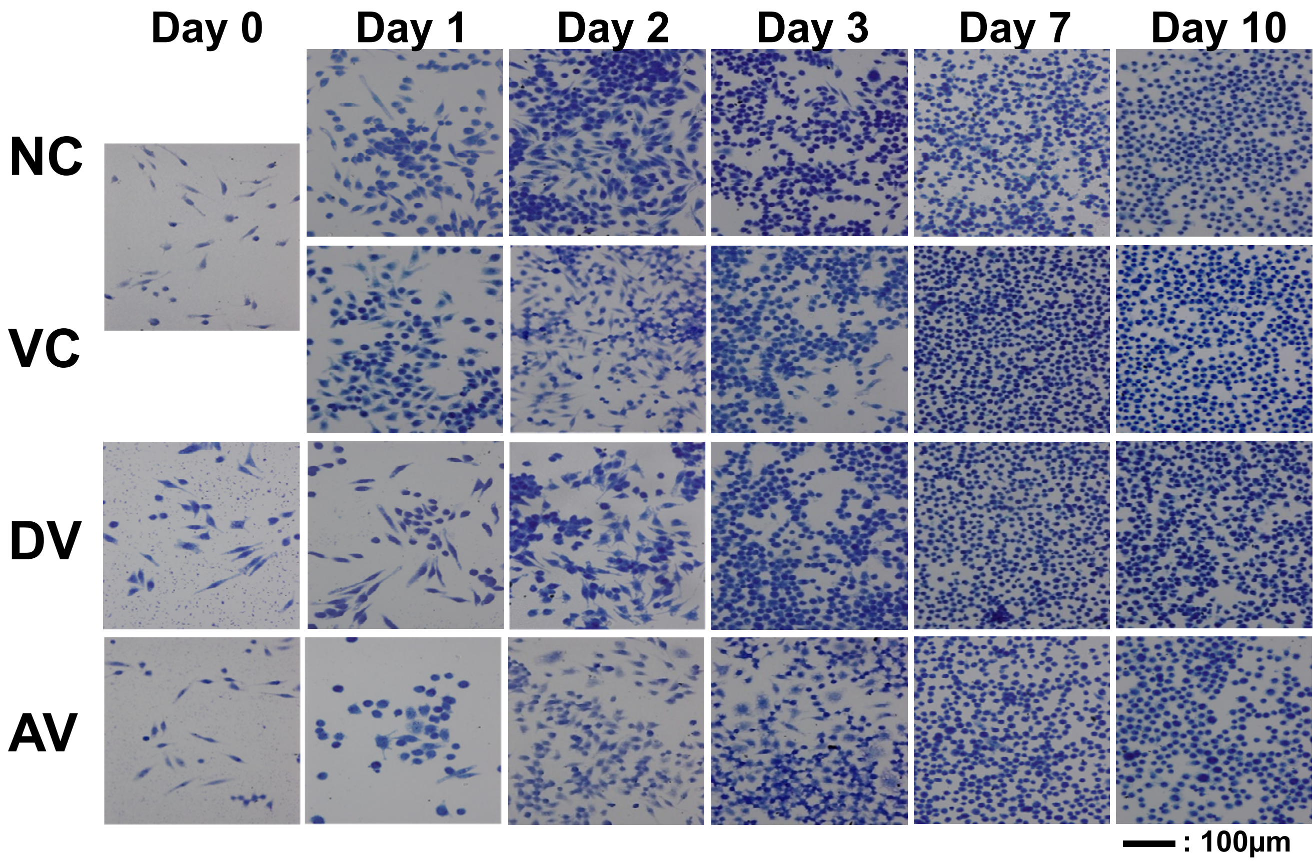

Figure S2. Dead/Alive in J774A with/without OJ-1 ingestion.

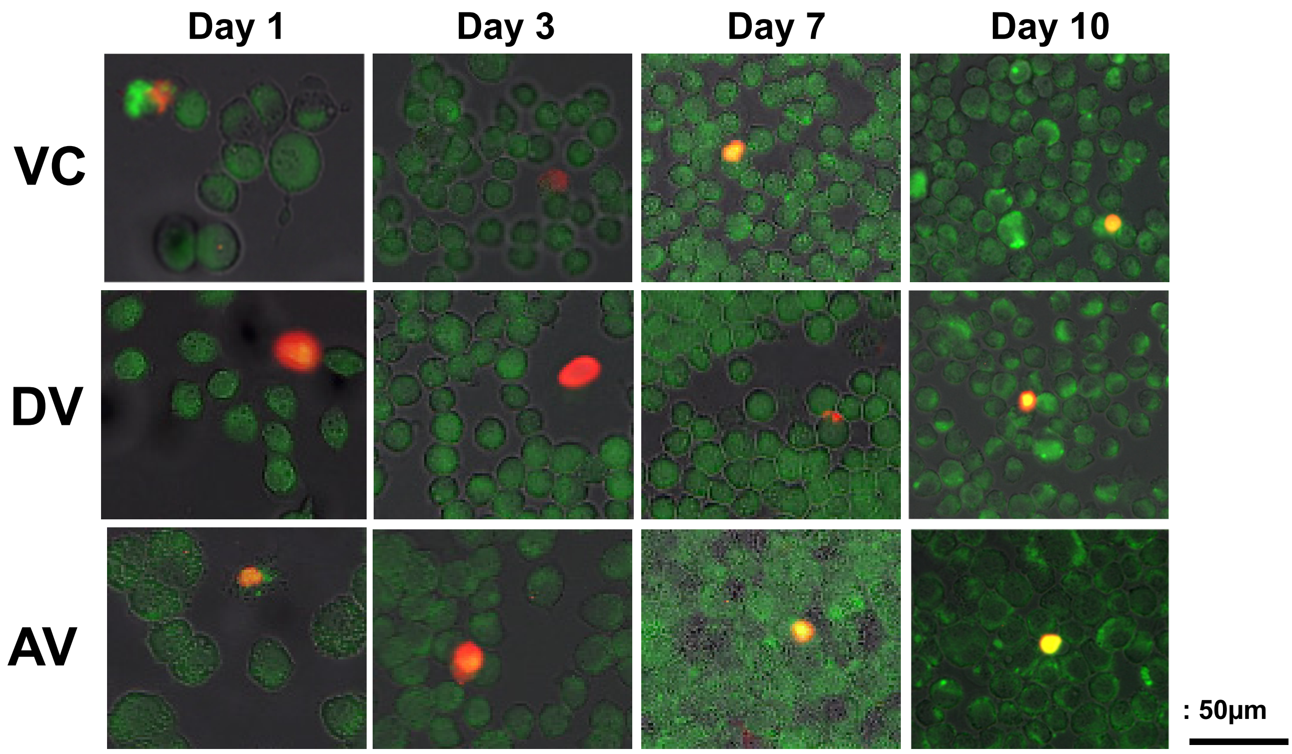

Supplement: Supplementary file 1 [file microorganisms-10-00348-s001.zip › microorganisms-1570732-supplementary.pdf]
